# “Marketing through Claims”: A Cross-Sectional Analysis of Child-Targeted Food Packaging Claims within the Spanish Market

**DOI:** 10.3390/nu15214548

**Published:** 2023-10-26

**Authors:** Alazne Arraztio-Cordoba, Maria Jose Montero-Simo, Rafael A. Araque-Padilla

**Affiliations:** 1 Department of Management, Universidad Loyola Andalucía, 41704 Seville, Spain; 2 Department of Management, Universidad Loyola Andalucía, 14004 Cordoba, Spain; jmontero@uloyola.es (M.J.M.-S.); raraque@uloyola.es (R.A.A.-P.)

**Keywords:** children, packaging, food, claims, marketing techniques, market

## Abstract

Food advertising, especially on packaging, impacts children’s choices. Food companies make different claims on packaging as a marketing techniques to make their products more appealing, enhancing their perceived healthiness, even in unhealthy products. Although the use of some claims is regulated, there are legal loopholes that could confuse young consumers and that concern global authorities. To shed light on the matter, it is necessary to have a comprehensive understanding of the performance of all types of claims made by food companies in food products targeting children. We examined 458 products from Spanish markets in two periods through a cross-sectional content analysis. Our findings reveal that existing policies are working due to the decrease in nutrition claims, but there is a growth in soft claims that are unregulated and potentially confusing and attractive to children. Considering that most of the products analyzed are unhealthy, this emphasizes the importance of implementing stricter regulations to create a healthier and more reliable food environment for children.

## 1. Introduction

Advertising can quickly capture people’s attention and significantly influence their preferences [1,2,3,4,5]. Aware of this power, food companies address much of their efforts to attract the attention of a vulnerable and easily influenced public, such as children [6,7,8,9]. One of the most widely used tools in pursuing that purpose is the packaging of food products. In an environment saturated with many options, the design and appearance of packaging can quickly, easily, effectively, and efficiently capture the attention of younger consumers thanks to the use of different marketing techniques such as cartoons, colors, serving suggestion images, promotions, or claims [10,11,12,13,14]. All of them interfere with the visual and aesthetic aspect of the product, making it more attractive and desirable, except for the claims, which are the only cues present on the packaging that directly allude to the healthiness of the product in which they are present.

According to European Commission (EU), a “Claim is any message or representation” “(…) which states, suggests or implies that a food has a particular characteristic” [15], drawing attention to the nutritional qualities or alleging health benefits of certain products. Considering that children can understand the importance of healthy eating [16], these messages can influence their perception of the healthfulness of a product [17], affecting their choices [18,19]. The risk comes when we realize that sometimes these claims are present in healthy foods but also unhealthy foods, generating some confusion or false expectations about a food product in the consumer [20,21,22]. Proper food choices are essential in following a healthy diet, and these claims could hinder these healthy choices, promoting the increase in the already high rates of childhood obesity in Europe [23].

To try to avoid this, and in order to ensure that food claims are accurate and verifiable, the European Commission and other institutions have established regulations controlling the use of certain types of claims [15,24]. In addition, both at the global and national levels, there are self-regulatory codes that seek to mitigate these practices, such as the Codex Alimentarius [25] or PAOS code in Spain [26]. It should be added that these regulatory systems focus their efforts on nutrition and health claims, requiring them to be based on scientific evidence for their formulation. As a result, there are other types of claims that, since they cannot be based on such evidence, are not regulated; claims that are also more appealing and effective, a fact that food companies take advantage of to continue making use of them and, therefore, continue to capture the attention of children [22,27,28,29]. Moreover, these regulations fail to consider or restrict foods that may or may not carry such claims, disregarding the fact that unhealthy food products today can still be advertised with such claims, potentially hiding any nutritional deficiencies. This means there is a situation of confusion and lack of protection for the consumer, thus hindering the performance of an enabling food environment, which is beginning to worry public authorities, different health organizations, and stakeholders [22]. Although some steps forward are being attempted in this regard [30,31], there is still a long way to go [29].

So, to shed light on the matter and to try to pave the way for these social agents, it is first essential to have a comprehensive understanding of the performance of these claims in packaging. Therefore, in this article, we pursue the following main research objective: to understand how the use of claims on food products’ packaging by food companies as a marketing strategy is evolving. And, more specifically, we propose the following research objectives:

1. To determine if there has been a shift in the number of claims in general and by type over time.
2. To determine if there has been a shift in the number of food products using claims over time.
3. To find out if there has been a shift in the use of claim usage in relation to product healthiness over time.

Moreover, it is worth noting that, although the analyses are conducted with a sample of products, the results could reflect what is happening in most of the products on the market.

## 2. Materials and Methods

We conducted a cross-sectional content analysis on 458 products collected in two separate periods, 2018 and 2022. Each year, we collected 229 products. To achieve our research objectives, we conducted different frequency analyses. Please see below for a detailed explanation of the methodology followed.

### 2.1. Selection of Supermarket

The data was collected from the Spanish market in 2018 and 2022. We started by visiting the leading international supermarket chains (Carrefour, Aldi, Lidl, and DIA). To expand the sample of products analyzed in the study, and after reviewing all products aimed at children in international supermarkets, it was decided to visit two national supermarket chains (Mercadona and Hipercor). Finally, to make the product review as exhaustive and complete as possible, it was decided to visit one last local supermarket (Deza). When we observed that we were no longer finding new products because they were beginning to repeat themselves in our database, we decided to end the supermarket visits and sample collection. In the end, a total of seven supermarkets were analyzed.

### 2.2. Sampling and Units of Data

The type of sampling used was a convenience sample that was non-probabilistic and non-random. The data units were “child-oriented” food products. To decide whether products were child-oriented or not, inclusion and exclusion criteria were determined according to both protocols for monitoring food marketing to children [32,33] and other authors [34,35,36,37]. To be selected, food products had to meet at least two of the following inclusion criteria in its packaging: (1) words that allude to children, such as “fun”, “games”, “physical activity”, or “school”, (2) images with characters, celebrities, influencers, children, or images that appeal to children, (3) emphasis on unusual shapes, unconventional flavors in adult products, or bright colors, (4) promotions or links to children’s TV programs, merchandising, films, and websites, and (5) premium offers such as games or toys. Boxes with the same product were registered as a single item; baby foods such as porridge, mashed fruit, purees, or milk formulas were excluded; seasonal products, such as Christmas, Easter, or Halloween, were excluded; products with slight variations in content (e.g., strawberry or banana flavored yogurts) were recorded as a single item; products of the same brand with the same nutritional content, but with variations in packaging were registered as a single item.

**Figure 1:**
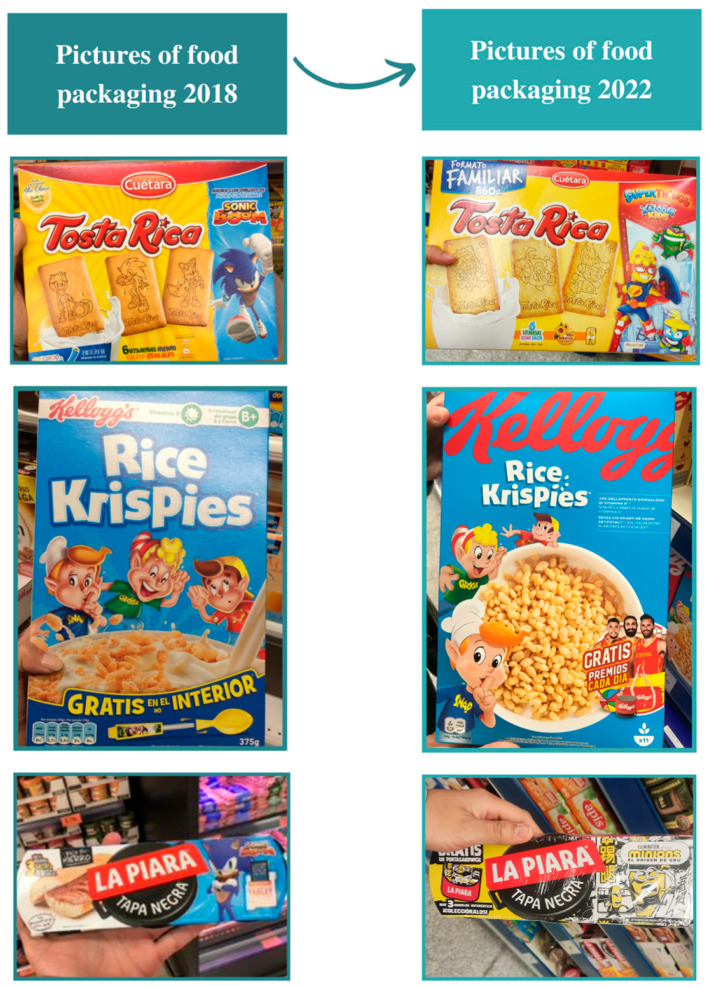
Photos of the food packaging of the products in 2018 vs. 2022.

In the first data collection in 2018, all products available in the analyzed supermarkets that met the inclusion criteria were photographed, regardless of their product category. Images were taken of the four sides of the products to encode the information afterward. In the second data collection in 2022, the same products, previously collected in 2018, were photographed, as seen in Figure 1. Most of the products selected belong to the main groups of the food industry. Some of these groups are as follows: Nestlé (Vevey, Swiss), Unilever (Englewood Cliffs, NJ, USA), Mondelez International (Chicago, IL, USA), General Mills (Minneapolis, MN, USA), Pepsico (Plano, TX, USA), Danone (Barcelona, Spanish), Bimbo (Barcelona, Spanish), among others. This implies that the perspective offered in this study can be extended to other countries, especially in the European context. Seven food product categories were finally identified, beverages, sweets, breakfast cereals, candies, savory snacks, processed meat, poultry, fish and similar, and dairy products. These food product categories align with the ones proposed by the World Health Organization in the Europe nutrient profile model [38]. Some products, such as those derived from chocolate or cocoa and ice creams, were classified as sweets due to their high sugar levels because they could generate ambiguity and to follow the classification of Davidović et al. [39] based on the FoodEx2 food classification system [40]. The descriptions of the food categories included in the database of 2018 and 2022 are presented in Table 1. No food products were found that meet our inclusion criteria that belong to other food categories.

### 2.3. Data Collection

The following information was subsequently extracted according to the photos of the food products: (1) product identification data (name of the product and name of the product brand), (2) nutrition labelling (nutrition facts, also known as FACTS), and (3) claims.

### 2.4. Types of Claims

**Figure 2:**
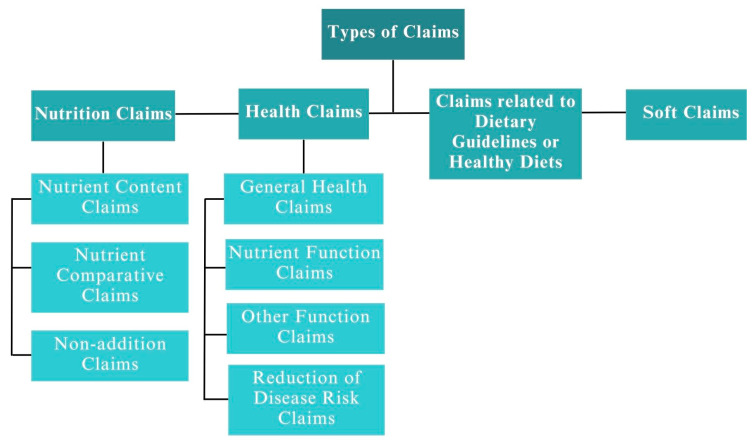
Types of claims.

To carry out this study, the claims extracted from the food products required a classification according to their typology, as seen in Figure 2. The proposed classification is based on current laws and regulations [15,24,25], including soft claims, to conduct a thorough and comprehensive analysis of all types of claims in food products.

To be able to classify claims correctly, the types of claims are defined as follows:

- Nutrition claim: “It is any representation which states, suggests or implies that a food has particular nutritional properties including but not limited to the energy value and the content of protein, fat, and carbohydrates, as well as the content of vitamins and minerals” [25]. Nutrition claims can be classified as one of the following three types:
  - Nutrient content claim: “This is a nutrition claim that describes the level of a nutrient contained in a food” (e.g., source of fiber) [25].
  - Nutrient comparative claim: “A claim that compares the nutrient levels and/or energy value of two or more foods” (e.g., increased calcium) [25].
  - Non-addition claims: “Any claim that indicates an ingredient that has not been added to a food, either directly or indirectly” (e.g., no added dyes) [25].
- Health claim: “Is any representation that stages, suggests or implies that a relationship exists between a food or a constituent of that food and health” [25]. Health claims can be classified as one of the following four types:
  - General health claim: “This is a claim which by definition refers to general, non-specific benefits of the nutrient or food for overall good health or health-related well-being” (e.g., healthy, fit, heart symbol) [39].
  - Nutrient function claim: “This is a claim that describes the physiological role of the nutrient growth, development, and normal functions of the body” (e.g., calcium is needed for the maintenance of normal bones) [25].
  - Other function claim: “Is a claim that concerns specific beneficial effects of the consumption of food or their constituents, in the context of the total diet on normal functions or biological activities of the body” (e.g., vitamin B6 contributes to normal psychological function) [25].
  - Reduction of disease risk claim: “This is a claim relating the consumption of a food or food constituent, in the context of the total diet, to the reduced risk of developing a disease or health-related condition” (e.g., a healthful diet low in sugar may reduce the risk of diabetes) [25].
- Claims related to dietary guidelines or healthy diets: “This is a claim related to the pattern of eating contained in dietary guidelines officially recognized by the appropriate national authority” (e.g., eat plenty of cereals, preferably wholegrain) [25].
- Soft claim: These claims offer a more holistic view of how healthy a product can be, or appeal to its healthiness indirectly or vaguely. Contrary to the other claims explained so far, these types of claims do not require the benefits of a product or a nutrient on the population’s health to be scientifically proven. It is precisely this fact that can cause consumer confusion or potentially mislead them (e.g., grandma’s traditional recipe) [27,41].

### 2.5. Assessing the Healthiness of a Product

**Figure 3:**
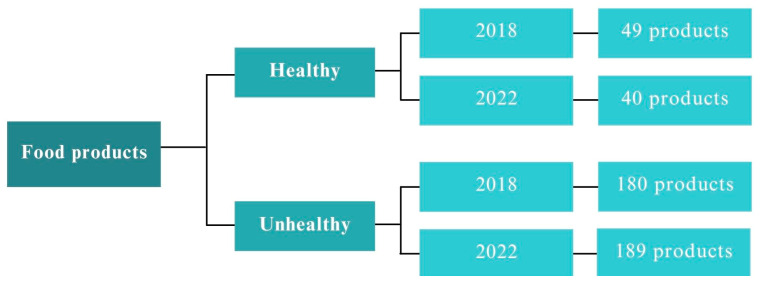
Changes in the number of healthy and unhealthy products in 2018 and 2022.

The analyzed foods were classified as healthy and unhealthy according to the information available on the nutrition FACTS label and the results obtained from the nutritional index. The index used is the OFCOM nutritional index, which manages to provide a single score (healthy or unhealthy) to foods and beverages depending on their values, energy, saturated fat, sugars, and sodium on one hand, and the amount of protein, fruit, vegetables, and nuts on the other. The model uses a measurement of 100 g as the basis for the calculations, considering that this quantity is legally used in the nutrition FACTS label and used to compare products with each other [42]. It is important to mention that some products that were considered healthy in 2018 may have become unhealthy in 2022. Therefore, Figure 3 provides information on the number of products that have undergone changes in formulation.

### 2.6. Data Collection Period

Data was collected throughout January 2018 and throughout March 2022.

### 2.7. Codification

In order to gather information from the photographs, a group of variables was carefully coded by three coders. The coding was completed with the intention of minimizing errors and ensuring thoroughness. The coders were recruited in June 2022. The coders coded all the food products in the sample for analysis and their findings were reviewed during regular meetings. Due to the mathematical calculations, the variables related to the classification of the OFCOM nutritional index required prior training. The variables had an initial agreement of 92% and the discrepancies were discussed (mostly related to OFCOM calculation errors) until a 100% agreement was reached. The coding of variables was finalized in July 2022.

### 2.8. Data Analysis

The data were analyzed with the statistical program software for data science Stata/SE 17.0. The statistical methods used are mean difference analysis and the chi-squared test (X2).

## 3. Results

458 products targeted at children from 7 food categories were collected, 229 were collected in 2018, and the remaining 229 products were collected in 2022, looking for the latter to be the same as in 2018 to make appropriate comparisons. The following paragraphs will explain the research results, focusing on answering our research objectives. In this way, we want to understand the trends in the use of claims by analyzing the evolution of their use by the food industry in the Spanish market.

**Figure 4:**
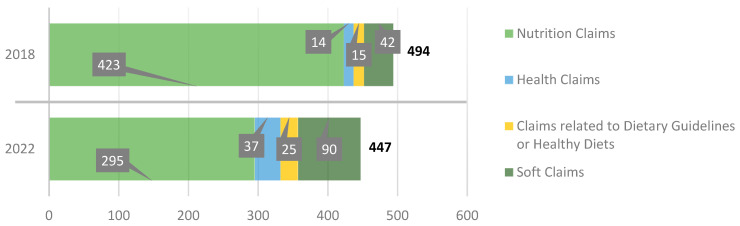
Evolution of the number of all types of claims.

**Figure 5:**
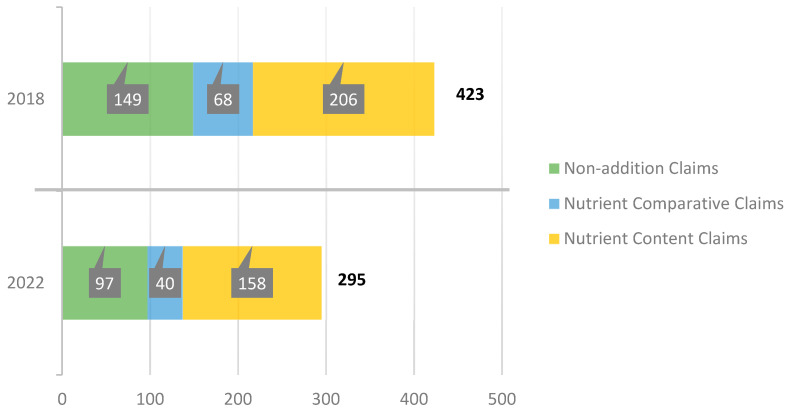
Evolution of the number of all types of nutrition claims.

Overall, we observed a slight decrease in the total number of claims used and presented on the packaging of food products aimed at children by the food industry in Spain during the period of analysis. However, if we look at the different types of claims, we see that this decrease has only occurred in nutrition claims and their different subtypes: nutrient content claims, nutrient comparative claims, and non-addition claims. However, all other types of claims have increased, including the health claim subtypes (Figure 4 and Figure 5).

If we examine food products aimed at children with some type of claim on the packaging, we can draw different findings. The number of these products has decreased over time (76.4% in 2018 vs. 64.6% in 2022, *p* = 0.005). Nevertheless, if we attend to the usage ratio, this decline is not replicated; the average number of claims per product raised (2.83 claims per product in 2018 vs. 3.25 in 2022). This implies a higher use of claims among most products over time (84.58%). Id est, most products have maintained and increased their use of claims over the years analyzed. These results, however, varied concerning the different types of claims.

There was a decrease in the number of food products with nutrition claims on their packaging (72.05% in 2018 vs. 56.76% in 2022, *p* = 0.001), a decrease which is also reflected in the usage rate (2.56 claims per product in 2018 vs. 2.26 in 2022). When considering the different types of nutrition claims, we observed that only nutrient content claims yielded significant results, with few products using those claims on their packaging. However, the ones that did use those claims used them more frequently (0.47 claims per product in 2018 and 0.58 claims per product in 2022). Examples of this type of claim collected in the study are listed as follows: “With 10 vitamins” or “With vitamin D, vitamin B6, and calcium”.

Upon examining the recent use of health claims, a noticeable trend can be observed compared to previous claim types. The number of products using health claims that target children has increased, as well as the frequency of their usage per product. In 2018, the average usage of health claims per product was 1.4; by 2022, it had risen to 1.76. It should be noted that a few products have been collected with claims from the health claims subgroups. General health claims, other function claims, and reduction of disease risk claims do not allow us to draw accurate conclusions, except for nutrient function claims, in which a significant growth rate in their use by food products (2.2% in 2018 and 6.5% in 2022, *p* = 0.019) was observed. Below are some examples of the nutrient function claims collected in this study: “Niacin contributes to normal energy metabolism” or “Vitamin D contributes to the maintenance of normal bones”.

Focusing on claims related to dietary guidelines or healthy diets, we observed no significant outcomes due to the limited number of products with such claims on their packaging.

In contrast, soft claims have become increasingly common in food products, with the appearance rate doubling over the years. However, the usage rate has slightly decreased from 1.75 claims per product in 2018 to 1.60 in 2022. A classification has been created based on their content to enhance the understanding and targeting of these messages. Please refer to Table 2 for more information.

It should be noted that, in general, there was a decrease in the number of claims for healthy and unhealthy products; however, this reduction is much more pronounced for healthy products, as they have 34% fewer claims in 2022 compared to 2018. By contrast, the number of claims for unhealthy products reduced almost imperceptibly by 2% in 2022 compared to 2018.

**Figure 6:**
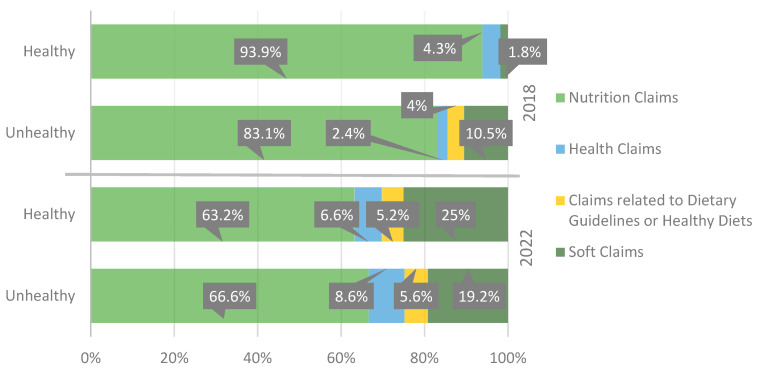
Evolution in the distribution (percentage) of all types of claims according to the healthiness of the food products.

If we attend to the different types of claims analyzed in this study, as shown in Figure 6, we can see that there is a reduction in the use of nutrition claims, both in healthy and unhealthy products over time. Health claims and claims related to dietary guidelines or healthy diets increase slightly in both healthy and unhealthy products. The significant increase in the use of soft claims in unhealthy, and very evidently, in healthy products is worth noting.

**Figure 7:**
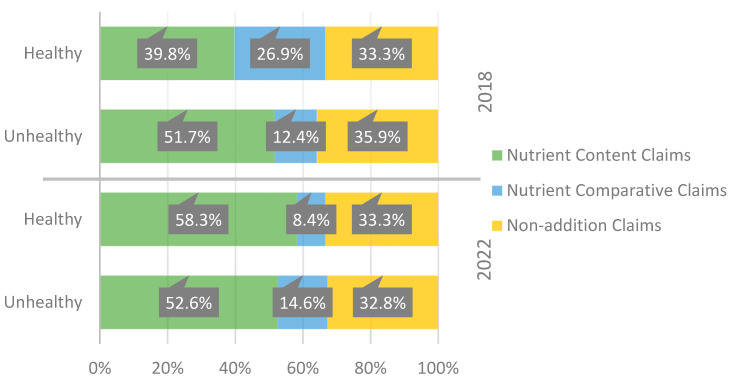
Evolution in the distribution (percentage) of nutrition claims according to the healthiness of the food products.

As seen in Figure 7, the food industry has been using nutrition claims, nutrient content claims, nutrient comparative claims, and non-addition claims less frequently in food packaging for children from 2018 to 2022 in both healthy and unhealthy products. Despite this reduction, its distribution across the different food products that have nutrition claims has not changed by much over time.

No significant changes were found in the rest of the claims subtypes concerning the healthiness of food products.

Focusing on the different food categories, we can point out that those with the unhealthiest products remain stable over the period of analysis. These are sweets, candies, savory snacks, and breakfast cereals.

Notably, the food industry decreased the use of nutrition claims in the sweets category (*p* = 0.030), but also significantly increased the use of soft claims in the same category (*p* = 0.05), with this food category having the highest number of unhealthy products in our sample.

Moreover, there is a decrease in the use of nutrition claims in processed meat, poultry, fish, and similar food categories (*p* = 0.000) and in Dairy products (*p* = 0.000). These categories do contain reasonably healthy food products in them; in fact, dairy products improve their level of healthiness by 17.08% in 2022 compared with 2018.

For the rest of the food categories, no statistically significant differences are found with respect to the claims used on their packaging, except for breakfast cereals, where the presence of nutrient function claim increases (*p* = 0.019).

## 4. Discussion

To our knowledge, this is the first study to comprehensively analyze how food companies have been using all types of claims on the packaging of food products aimed at children, and therefore, that presents knowledge of the trends in the use of marketing techniques in the food industry. The findings obtained will be discussed below.

Firstly, it is worth mentioning that the nutritional quality of the products we have analyzed has not improved over time. Therefore, within the products analyzed, there has not been a concern on the part of the food industry to make them healthier according to the OFCOM nutritional index. These findings, apparently worrying, are similar to those found in a study also conducted in Spain [43] and to other European countries such as France [44], Belgium [45], or Slovenia [46], and also worldwide in countries like the United States [47], Canada [22], Uruguay [36], Guatemala [35,48], Brazil [49], Australia [50], and New Zealand [51]. According to the recommendations put forth by the World Health Organization (WHO) Regional Office for Europe’s nutrient profile model [31], it is evident that food companies should not promote unhealthy products through any form of marketing strategy. However, our study reveals that the current state of food products targeting children in Spain does not align with this ideal. Regardless of their nutritional value, most food items showcase a range of claims on their packaging, accompanied by other marketing techniques. Below, we comprehensively analyze the behavior of these claims based on the examined time period and food product sample and draw conclusions.

Before discussing the first and second research objectives, it is important to note that, apparently, food companies in Spain have reduced the usage of claims on product packaging aimed at children over the years, however, this slight downward trend has not been replicated in the same way for all types of claims or foods. The most used types of claims in food products aimed at children by food companies are nutrition claims (particularly nutrient content claims), soft claims, and health claims. In our analyses, we have found two trends of change over time. The first is the general decrease in the use of nutrition claims in the products analyzed, both in the total number of nutrition claims in the sample, and in the number of products in which they appear and the number of times they appear per product. This trend is also evident in the nutrient content claim subgroup, but only in the number of products that use them. The second trend we have found in our study—a shift in the opposite direction to the previous claim explained above—is the growth in the use of soft claims, health claims, and claims related to dietary guidelines or healthy diets by the products analyzed. In the case of soft claims, this increase has been particularly relevant.

From these results, we can draw three main and relevant conclusions. The first one is the effectiveness and usefulness of public policies, especially with nutrition claims, but not so much with health claims. This can be seen in the way that food companies have been gradually adapting to the European regulations and in the way that they are decreasing the use of nutrition claims, as has already happened in Canada after the reform of its regulations related to nutrition labeling by the government [52], or in another study conducted in Spain focusing on products that are not aimed at children [29]. As for the increase of health claims, despite their reduced presence, there is an increase in their appearance in food products, even though they are equally regulated and aligned with nutrition claims, whose regulation is, in fact, included in the same policy.

The third conclusion is that these food companies, taking advantage of the fact that soft claims are not yet regulated, have greatly increased their use in food products. This may be due to the increased regulation of other marketing techniques such as those mentioned above. Soft claims have been shown to create a positive perception of the product, especially those that refer to the naturalness and tradition of the product, to fun, or even to health [27,53]. This could imply that such products, by regulation, that can no longer use nutrition or health claims could use soft claims to continue attracting the attention of children.

This is undoubtedly worrying because, as our analyses have revealed, it is precisely in the categories of foods with less healthy products, such as sweets, where the phenomenon we have just mentioned has occurred the most since the presence of nutrition claims in these products has decreased significantly (taking into account the nutritional value of the products that are part of this category). However, it is also the category of foods in which the use of soft claims has increased the most. Indeed, the level of healthiness of these products should not sustain the presence of nutrition claims. Nevertheless, due to the lack of regulation affecting soft claims, there is nothing to prevent their use as a marketing tool by food companies [54].

We should not always look at soft claims as inciting agents for the consumption of unhealthy foods, especially considering what has happened with the healthy products in our sample over time. We have been able to observe that it is precisely in healthy products where the increase in soft claims has been most marked. At the same time, it is also in the healthy products where there has been the greatest decrease in the use of nutrition claims by food companies. On the one hand, the increase in soft claims for this type of product may have been caused by the desire to attract children to healthier foods. On the other hand, the decrease in nutrition claims on healthy products may be causing food companies to miss the opportunity to promote healthier foods not only to children, who are increasingly concerned about their diet, but also to parents, who may better understand the purpose of these claims.

This leads us to understand precisely why agents such as the European Commission, on one hand do not prohibit soft claims, and on the other hand, according to the farm-to-fork strategy [55], insist on the creation and implementation of nutritional profiles focused on the nutritional quality of food to restrict the presence of marketing in unhealthy foods aimed at children, reducing their consumption [56], but not prohibiting its use in healthy foods, since, soft claims, or any other type of marketing technique, can be a good tool to increase the consumption of healthy foods by children [18,57], and even influence their dietary behavior [58]. Countries such as the United Kingdom, Brazil, Canada, Chile, and Peru are already implementing them [59] and are interested in reaching a worldwide consensus.

### Strengths and Limitations

Our study presents a comprehensive evaluation of the evolution in the trend of using claims by food companies within the Spanish market. This research, facilitated by the employed data collection methodology, captured a significant number of products targeted at children in the Spanish market. The analyses were not limited to specific food categories or particular types of claims, ensuring a broader scope of examination. Also, by employing electronic devices to capture images of all food items in the store, we were able to gather and process data in a highly efficient manner and in an easily replicable form. Our study is the first, as far as we know, to examine products aimed at children within the Spanish market, laying the groundwork for future studies to continue research in this area. Furthermore, the inclusion of products of international origin in our analysis has yielded results with extrapolation potential beyond Spain. Approximately 70% of the products examined in our study were sourced from international origins, thereby enhancing the generalizability of our findings.

Nevertheless, our study does have certain limitations that should be acknowledged. First and foremost, we lacked a pre-existing database compiled prior to the European Commission regulations, which would have enabled a conclusive prior analysis and provided comparative results to enhance the comprehensiveness of our study. Second, due to our desire to compare the same products in both years to make as exhaustive a comparison as possible, we have not analyzed new products launched in 2022 aimed at children that did not exist in 2018. This could, in some way, have limited our results, and should be taken into account in upcoming studies. For future research, it would also be interesting to investigate future trends in health claims, given that we have been able to observe their increase, but only on the basis of the reduced number of claims present in the products analyzed. Furthermore, conducting a comparative analysis with other European Union countries would be of great interest, aiming to observe whether the legislation in force has yielded similar effects in those markets as well. Achieving this objective would require standardization in terms of sampling and data collection, but the insights that could be derived from such an endeavor would undoubtedly be highly valuable.

## 5. Conclusions

Despite the recommendations made by the World Health Organization, our research findings show that food companies are still promoting unhealthy food products to children [31]. However, we have noticed that these companies have adapted their targeting strategies over time. We have identified two specific trends. A reduction in the use of nutrition claims and an increase in the use of soft claims. Apparently, companies are adjusting their marketing strategies in response to current European regulations, taking advantage of the lack of regulation on soft claims.

Nevertheless, these trends have not manifested themselves equally across healthy and unhealthy products. While food companies have drastically reduced their use of the total number of claims on healthy products, the prevalence of claims on less healthy products has remained almost unchanged. This leads us to a relevant conclusion: despite being aware of the power of claims, the strategies of food companies have not proved to be adequate in fostering healthier food habits in children. They seem to prioritize the promotion of less healthy options instead of focusing on promoting healthier alternatives. This undoubtedly hinders progress toward promoting healthier dietary choices among consumers, especially children, and hinders the improvement of our current food environments.

## Figures and Tables

**Table 1:** Descriptions of the food categories included in the database of 2018 and 2022.

Product Category	Description
Beverages	Products including juices (100% fruit and vegetable juices or reconstructed from concentrate, smoothies), lemonade, orangeade, other soft drinks, and mineral or flavored waters.
Sweets	Cakes, sweet biscuits, pastries, other sweet bakery wares, fruit pies, chocolate, and other products containing cocoa and ice creams.
Breakfast cereals	Oatmeal, cornflakes, chocolate breakfast cereals, mueslis.
Candies	Jelly, chewing gum and bubble gum, caramels, and licorice sweets.
Savory snacks	Popcorn and maize corn, seeds, nuts and mixed nuts, savory biscuits and pretzels, and other snacks made from rice, maize, dough, or potato.
Processed meat, poultry, fish, and similar	Sausage, ham, bacon, chicken nuggets, smoked and pickled fish fish fingers, and breaded/battered fish.
Dairy products	Yogurts, sour milk, creams, grated or powdered cheese, cheese-based products, and milky drinks.

**Table 2:** Types of Soft Claims analyzed.

Soft Claims	Nº	Examples
Related to fun and play	55	“United by the power of friendship”, “Let’s shake it up, let’s get this party started”, “Flakes make me crazy”, “The happiest popcorn in the world”.
Related to food preparation, flavor, and tradition	48	“Crunch!”, “The snack of a lifetime”, “Prepared with care”, “Respecting the traditional recipe of the crêpe”.
Related to health	23	“Vital nutrition expert”, “Our children’s ice creams offer a choice that delivers flavour, fun and nutrition”, “Smart nutrition”, “They’ll eat breakfast for sure”.
Related to responsibility	6	“Created for kids”, “Quality for kids”, and “Responsibly made for kids”.
Total	132	

## Data Availability

Not applicable.
